# A Thing of Beauty

**Published:** 1875-10

**Authors:** 


### A THING OF BEAUTY.

For a year or more we have noticed a rare display of artistically constructed lamps in the window of the store, No. 23 Union Square, New York, and have been especially attracted to the vast volume of light pouring out of that window at night upon the passers by. We had observed the sign,—"Duplex Lamp"—too, but we did not comprehend its significance.

At the Institute Fair, we had an opportunity to examine some of these lamps, and we became greatly interested in them. We afterwards visited the store on Union Square, and saw a number of them in full operation, and have since practised reading and writing by their aid, together with other tasks which try the eyes. Now we feel qualified to speak intelligently concerning them.

To begin with, let us say that they are beautiful. There are choice bronzes, exquisite cut glass, delicate ormolu, porcelain artistically ornamented in many tints, and other chaste and attractive designs,—some massive, and some light and graceful. The taste finds ample scope for gratification among the hundreds of rich designs. There are also plainer styles for those who seek not decoration, and prefer severe simplicity.

A point of greater importance is that these lamps are safe. They do not conceal imminent danger under a fair exterior. They are honest, and can be trusted. They do not explode, as do cheap and poor kerosene lamps, nor do they clog up and smoke and behave viciously, and refuse to burn, and give off a long column of dense smoke instead, as do the German Student-Lamps, on frequent occasions. Their safety is assured by the mode of construction. A collar of non-conducting cork is arranged between the oil and the burner, which prevents the heating of the oil and the formation of gas in the lamp. Thus it is found possible in this lamp to use oils which, from their explosive tendency when heated, would be unsafe in any ordinary lamp. Cold kerosene, however volatile, never explodes; heated kerosene, if of a dangerously volatile character, explodes readily. A lamp which, when burning, heats its contents, is a dangerous lamp.

With the two features of elegance and safety established, it would seem that but little more could be said. With a lamp to please the eye of the most fastidious, and to be safe, even if used with oil usually deemed hazardous; with a lamp easy to manage and not inclined to smoke, what more can be asked?

There is one thing besides,—and that is the volume and quality of the light produced. This is the strong point in the Duplex Lamps. We have seen and used many lamps, but we are willing to testify that we have never before seen such illuminating power as this lamp possesses. It has two separate and distinct wicks, one or both of which can be burned, as desired. With one lighted, a very brilliant and pure white light is secured; with both ablaze, the light is intense, and probably unequalled by any portable light known.

These wonderful lamps are not made in America, but are the product of the best artizans of the old world. In England they are very widely used, even in localities cheaply provided with excellent illuminating gas, on account of the superiority and healthfulness of this light. That this lamp must come into very general, if not universal use, among refined people, we feel assured,—as will all, we imagine, who will examine its advantages as completely as we have done.

Mr. F. H. Simpson, General Agent for the Duplex Lamp Company, will take pleasure in exhibiting and explaining the lamp at 23 Union Square, New York.

